# Phage Lytic Protein LysRODI Prevents Staphylococcal Mastitis in Mice

**DOI:** 10.3389/fmicb.2020.00007

**Published:** 2020-01-23

**Authors:** Diana Gutiérrez, Victoria Garrido, Lucía Fernández, Silvia Portilla, Ana Rodríguez, María Jesús Grilló, Pilar García

**Affiliations:** ^1^DairySafe Group, Departamento de Tecnología y Biotecnología de Productos Lácteos, Instituto de Productos Lácteos de Asturias (IPLA-CSIC), Villaviciosa, Spain; ^2^Departamento de Sanidad Animal, Instituto de Agrobiotecnología, CSIC-Gobierno de Navarra, Mutilva, Spain

**Keywords:** endolysin, antimicrobial activity, Staphylococcus, mastitis, bacteriophages

## Abstract

Phage lytic proteins are promising antimicrobials that could complement conventional antibiotics and help to combat multi-drug resistant bacteria that cause important human and animal infections. Here, we report the characterization of endolysin LysRODI (encoded by staphylophage phiIPLA-RODI) and its application as a prophylactic mastitis treatment. The main properties of LysRODI were compared with those of endolysin LysA72 (encoded by staphylophage phiIPLA35) and the chimeric protein CHAPSH3b (derived from the virion-associated peptidoglycan hydrolase HydH5 and lysostaphin). Time-kill experiments performed with *Staphylococcus aureus* and *Staphylococcus epidermidis* demonstrated that the killing rate of LysRODI and CHAPSH3b is higher than that of LysA72 (0.1 μM protein removed 10^7^ CFU/ml of *S. aureus* in 30 min). Of note, all proteins failed to select resistant mutants as bacterial exposure to sub-lethal concentrations of the proteins did not alter the MIC values. Additionally, LysRODI and CHAPSH3b were non-toxic in a zebrafish embryo model at concentrations near the MIC (0.5 and 0.7 μM, respectively). Moreover, these two proteins significantly reduced mortality in a zebrafish model of systemic infection. In contrast to LysRODI, the efficacy of CHAPSH3b was dose-dependent in zebrafish, requiring higher-dose treatments to achieve the maximum survival rate. For this reason, LysRODI was selected for further analysis in mice, demonstrating great efficacy to prevent mammary infections by *S. aureus* and *S. epidermidis*. Our findings strongly support the use of phage lytic proteins as a new strategy to prevent staphylococcal mastitis.

## Introduction

Multidrug resistant (MDR) pathogenic bacteria cause nowadays more than 700,000 deaths per year in the world, affecting developing and developed countries, rural and urban areas, hospitals, farms and communities^1^. Since the emergence of MDR pathogenic bacteria is an urgent global risk that remains unsolved, it is imperative to strengthen antimicrobial use regulations and foster innovative research on new ways to combat bacteria. WHO has developed a global priority list of MDR pathogenic bacteria, in which *Staphylococcus aureus* is considered as Priority 2 (High), with the aim of helping to prioritize research and development of new antibiotic treatments^2^.

*S. aureus* is along with *Staphylococcus epidermidis* an important cause of bovine mastitis, the most widespread disease in dairy cattle (Nyman et al., 2018). These infections are very difficult to cure due to the ability of staphylococci (and *S. aureus*, in particular) to adhere and proliferate on animal tissues forming a biofilm that, together with somatic cells, clog milk canals within the mammary gland. This leads to an insufficient penetration of the antibiotic in the mammary gland when infused into the udder. Moreover, the increasing rate of MDR strains; and the ability of *S. aureus* to persist within mammary gland epithelial cells in a low metabolic state, thereby evading antibiotic treatment, is the main cause of recurrent and chronic infections associated to mastitis infections (Gomes et al., 2016). Apart from the derived economic losses, mastitis is also the main cause of antibiotic use in the dairy industry (Hogeveen et al., 2011). However, the increasing generation of MDR pathogenic bacteria is promoting a more restrictive use of antimicrobials in animal husbandry that is totally banned in some countries as prophylactic in animal feed (European Commission, 2017).

Over the last years, the study of bacteriophage-encoded lytic proteins, including endolysins and virion-associated peptidoglycan hydrolases (VAPGHs), for the treatment of infectious diseases has attracted great interest (Fischetti, 2010; Rodriguez-Rubio et al., 2016a). These enzymes kill bacteria through peptidoglycan degradation followed by osmotic lysis. A pipeline portfolio recently published in The Lancet Infectious Diseases, highlighted endolysin-based antimicrobials as the compounds with the greatest potential to be used as novel therapeutics (Czaplewski et al., 2016). Indeed, there is evidence that many phage lytic proteins (enzybiotics) are effective in animal models of infection (Schmelcher et al., 2015a) and also clinical phase I trials have been completed without the observation of adverse effects (Jun et al., 2017). Phage lytic proteins have also been proposed as potential disinfectants for the food industry (Gutierrez et al., 2016) due to their rapid bactericidal action and biodegradability.

Most endolysins from phages infecting Gram-positive bacteria have a modular structure (Oliveira et al., 2013), which facilitates protein engineering *via* domain shuffling to obtain new chimeric proteins with improved lytic activity (Blazquez et al., 2016). Regarding *S. aureus*, several published reports have shown the effectiveness of phage lytic proteins as therapeutics (Schuch et al., 2014), biopreservatives (Chang et al., 2017) and disinfectants (Gutiérrez et al., 2014).

In our previous work, we identified and characterized four *S. aureus* infecting phages, the siphophages phiIPLA88 and phiIPLA35 and the myophages phiIPLA-RODI and phiIPLA-C1C (García et al., 2009; Gutiérrez et al., 2015). Two phage lytic proteins were identified in phage phiIPLA88, the endolysin LysH5 and the VAPGH protein HydH5 (Obeso et al., 2008; Rodríguez et al., 2011). Endolysin LysH5 has showed lytic activity against *S. aureus* in pasteurized milk (Obeso et al., 2008; García et al., 2010) and also against staphylococcal biofilms (Gutiérrez et al., 2014). Moreover, chimeric proteins derived from HydH5 and the bacteriocin lysostaphin were also obtained by combination of domains. The chimeric protein CHAPSH3b (CHAP domain from HydH5 and the SH3b CBD from lysostaphin) showed the highest specific activity (Rodriguez-Rubio et al., 2012). Subinhibitory doses of this chimeric protein can prevent *S. aureus* biofilm formation through the downregulation of autolysin-encoding genes (Fernandez et al., 2017). Moreover, two novel endolysins from phages phiIPLA35 and phiIPLA-RODI (*orf60* and *orf57*, respectively) were identified (García et al., 2009; Gutiérrez et al., 2015).

In this work, we have compared the main properties, in terms of enzymatic activity, of three phage lytic proteins; the two novel endolysins LysRODI and LysA72, and the chimeric protein CHAPSH3b. In a first step, a selection of the highest active proteins was performed based on *in vitro* experiments. Then, they were further validated *in vivo* using a systemic infection model in zebrafish. Finally, the ability of the most active protein (i.e. LysRODI) to prevent staphylococcal-induced mastitis was analyzed in lactating mice.

## Materials and Methods

### Bacterial Strains and Culture Conditions

The staphylococcal strains used in this study (Table 1) were grown in tryptic soy broth (TSB; Difco, Franklin Lakes, NJ) at 37°C with shaking at 250 rpm or on TSB plates containing 2% (wt/vol) bacteriological agar (TSA). *Escherichia coli* BL21 (DE3) was used for protein expression grown at 37°C with shaking in LB medium (1% tryptone, 0.5% yeast extract, 1% NaCl) or on plates of LB supplemented with 2% (w/v) agar. For proper selection of the clones, 100 μg/ml of ampicillin (Sigma Aldrich, Madrid, Spain) were added.

**Table 1:** *Staphylococcus* spp. strains used in this work.

Specie	Strain	Origin	Reference	MIC (μM)^a^		
				**LysRODI**	**LysA72**	**CHAPSH3b**
*S. aureus*	Sa9	Milk from cows with clinical mastitis	García et al., 2009	0.57	1.47	0.33
	Sa10			0.57	1.47	0.33
	15,981	Human clinical isolate. Strong biofilm former	Valle et al., 2003	1.15	1.47	0.66
	ATCC^®^ 25923™	Human clinical isolate with the designation Seattle 1945 that is used as a standard laboratory testing control strain	ATCC strain	0.57	1.47	0.66
	7,829	Pig skin. Methicillin-resistant *S. aureus* (MRSA)	Unpublished	1.15	5.88	0.66
	Staph. 15	Human clinical isolate. Methicillin-resistant *S. aureus* (MRSA)	Unpublished	1.15	5.88	1.33
*S. epidermidis*	F12	Milk from woman with mastitis	Delgado et al., 2009	1.15	>23.52	0.33
	B			0.57	>23.52	0.33
	DG2n			1.15	>23.52	1.33
	LV5RB3	Milk from healthy woman	Delgado et al., 2009	2.29	>23.52	0.33
*S. sciuri*	101	Milk from healthy woman	Martín et al., 2012	4.59	>23.52	0.33
*S. hominis*	ZL31–13			1.15	>23.52	0.33
*S. pasteuri*	ZL16–6			2.29	>23.52	0.66
*S. xylosus*	ZL61–2			1.15	>23.52	0.66
*S. saprophyticus*	ZL112–15			0.15	>23.52	0.66
*S. arlattae*	ZL114–5			1.14	>23.52	0.66
*S. haemolyticus*	ZL89–3			0.57	1.47	0.33
*S. gallinarum*	ZL90–5			1.14	>23.52	0.33
*S. kloosii*	ZL74–2			1.14	>23.52	0.66

a *Minimum inhibitory concentration (MIC) expressed as μM*.

*Data represent the mode of three independent biological repeats*.

### Structural and Functional Bioinformatics Analysis of the Endolysins

The protein sequences of LysRODI (Genbank accession number YP_009195893.1) and LysA72 (Genbank accession number YP_002332423.1) were analyzed using BLASTP (Altschul et al., 1990) and HMMER v3.2.1^3^ in order to identify the enzymatic activity domains. The protein sequences were compared with those from staphylococcal phage endolysins available in public databases (Pubmed, accessed January 2019) by CLUSTAL W alignment using Geneious 7.1.3^4^. The 3D structure of the proteins was predicted using the web-based server Phyre2 (Kelley et al., 2015).

### Plasmid Construction and DNA Manipulation

The genes encoding LysRODI (Gene ID: 26623165) and LysA72 (GeneID: 7057022) were optimized based on *E. coli* codon usage by the OptimumGene™ codon optimization technology^5^. Additionally, *Nd*eI and *Xho*I restriction sites were added at the 5′ and 3′ end, respectively. The optimized sequences were synthetized and cloned into the pET21a vector by GenScript (Township, NJ, USA). This vector introduces a C-terminal His_6_-tag and carries an ampicillin resistance gene. The pET21a vector containing the gene was then transformed into *E. coli* BL21 (DE3). The clones overexpressing CHAPSH3b were obtained in a previous study (Rodriguez-Rubio et al., 2012).

### Protein Overexpression, Purification and Endotoxin Removal

Protein expression was performed by induction with 1 mM IPTG (isopropyl-ß-D-thiogalactopyranoside) and incubation at 16°C, 16 h, as previously described (Gutierrez et al., 2015). For control purposes for *in vivo* experiments, *E. coli* BL21 cells transformed with the empty pET21a vector were used and purified as the rest of the proteins (MOCK control). After protein expression, cells were centrifuged (10,000 rpm, 4°C, 15 min) and suspended in 10 ml of lysis buffer (20 mM NaH_2_PO_4_, 500 mM NaCl, 10 mM imidazole, pH 7.4). Pellets were freeze/thawed three times at −80°C. Sonication was carried out afterwards (15 × 5 s pulses with 15 s recovery on ice; 40% of amplitude). Then, the suspension was centrifuged at 10,000 rpm, 4°C, 30 min. Proteins were purified by immobilized metal ion affinity chromatography using nickel-NTA Superflow resin columns (Qiagen, Valencia, CA, USA) following supplier’s recommendations. Briefly, 1 ml of resin was added to 10 ml of protein lysate. This mixture was incubated at 4°C for 16 h with slow shaking and then loaded onto 5 ml columns (BioRad, Hercules, CA, USA). For purification and endotoxin removal, a protocol described previously was performed (Reichelt et al., 2006). When using the protein for *in vivo* experiments (endotoxin-free), columns were washed with 50 ml of lysis buffer supplemented with 0.1% Triton X-114. Afterwards, columns were washed with 20 ml of lysis buffer and 50 ml of wash buffer (20 mM NaH_2_PO_4_, 500 mM NaCl, 20 mM imidazole, pH 7.4). For all other experiments, Triton X-114 was omitted and columns were washed with 30 ml of lysis buffer and 50 ml of wash buffer instead. Finally, the purified proteins were recovered with 1.5 ml of elution buffer (20 mM NaH_2_PO_4_, 500 mM NaCl, 250 mM imidazole, pH 7.4) and subsequently stored at −80°C in the presence of 30% glycerol to prevent precipitation.

Protein purity was evaluated in 12% (vol/wt) SDS-PAGE run at 150 V using Criterion precast gels (BioRad), and further revealed *via* conventional Coomassie staining. Protein concentration was quantified by the Quick Start Bradford Protein assay (BioRad).

Prior to the experiments, the buffer was exchanged to 50 mM sodium phosphate (NaPi) buffer (pH = 7.4) using “Zeba™ Spin Desalting Columns, 7K MWCO, 5 ml” (Thermo Fisher Scientific, Madrid, Spain) following the supplier’s recommendations. Finally, proteins were filtered (0.45 μm PES membrane filters; VWR, Spain).

### Quantification of Specific Lytic Activity

Turbidity reduction assay was performed as previously described (Obeso et al., 2008) using *S. aureus* Sa9 cells suspended in NaPi buffer (50 mM; pH = 7.4) and treated with two-fold dilutions of the purified proteins (0.02–50 μM). Results were expressed as specific lytic activity (ΔOD_600_ × min^−1^ × μM^−1^) (Donovan et al., 2006). To evaluate the effect of several cations on enzymatic activity, different salts (KCl, MgCl_2_, NaCl, MnCl_2_, ZnCl_2_, CaCl_2_) were added at a final concentration of 10 mM. The effect of temperature in protein activity was evaluated by incubating an aliquot of the protein for 30 min at different temperatures (40–90°C), followed by a turbidity reduction assay. In addition, the effect of pH on activity was tested by diluting (1:100) the proteins into Britton-Robinson buffer (150 mM KCl, 10 mM KH_2_PO_4_, 10 mM sodium citrate, 10 mM H_3_BO_3_) adjusted to pH 3 to 11. In this case, the turbidity reduction assay was carried out with the *S. aureus* Sa9 cells also suspended in the Britton-Robinson buffer. All experiments were performed in triplicate.

### Minimum Inhibitory Concentration (MIC) of Phage Lytic Proteins

The MIC of the proteins was determined in triplicate by the conventional broth microdilution technique in TSB (CLSI, 2015). The MIC was defined as the lowest protein concentration that inhibited visible bacterial growth after 24 h of incubation at 37°C and expressed as the mode of three replicates.

### Time-Killing Assays

The antibacterial activity was performed as previously described (Rodriguez-Rubio et al., 2016b) using as substrate *S. aureus* Sa9 diluted in TSB (~10^7^ CFU/ml) and 0.1 μM of each protein. As a control for bacterial growth, 50 mM sodium phosphate (NaPi) buffer (pH = 7.4) were added instead of the proteins. The cultures were incubated at 37°C with shaking (250 r.p.m) and 50 μl of samples were taken at different time points (2–60 min). Reaction was immediately stopped by adding 0.15 μg of proteinase K. Bacterial dilutions were then plated onto TSA and incubated at 37°C for 16 h. The antibacterial activity of three independent replicates was quantified as the relative inactivation in log units [log_10_(*N*_0_/*N*_i_) with *N*_0_ as the initial number of untreated cells and N_i_ as the number of residual cells counted after treatment] (Rodriguez-Rubio et al., 2016b).

### Development of Bacterial Resistance to Lytic Proteins

Resistance development was tested using repeated exposures to protein concentrations according to the minimal inhibitory concentration (MIC) assays (Becker et al., 2016). Two-fold serial dilutions of the proteins (0.02–50 μM) were used against *S. aureus* Sa9 (10^6^ CFU) and *S. epidermidis* F12 (10^6^ CFU) grown in a 96 polystyrene microtiter plate at 37°C for 16 h. The next day, 100 μl of the first well with growth (0.5 × MIC) were inoculated into 5 ml of TSB and grown to an OD_600_ = 0.5. These cultures were used for the next round of MIC exposure. The experiment was repeated for 10 rounds, followed by 5 additional rounds in TSB without the protein in order to allow phenotype reversion. Then MIC assay was further performed to measure the sensitivity to the proteins. Experiments were performed using three independent biological replicates. As a positive control of the experiment, the bacteriocin lysostaphin (Sigma, Missouri, USA), produced by *Staphylococcus simulans*, was also tested.

### Biofilm Assays

Staphylococcal biofilm formation and removal was performed as previously described using the RTCA technology (Gutiérrez et al., 2016; Gutierrez et al., 2017). Briefly, 100 μl of *S. aureus* 15,981 were diluted in TSBg (TSB supplemented with 0.25% w/v D-(+)-glucose) and poured into 16-well E-plates (∼10^6^ CFU/well). E-plates were connected to the xCelligence RTCA-DP (ACEA Biosciences Inc., San Diego, CA, USA) holder which measure the impedance signal and represent cell index (CI) values. Biofilm were formed for 8 h at 37°C and then 100 μl of LysRODI and LysA72 were added to achieve a concentration gradient (0.14–9.15 μM and 0.21–13.42 μM, respectively) and incubated for an extra 16 h. Impedance values were further processed using the RTCA software 1.2.1 (ACEA Biosciences Inc.) as described previously (Gutierrez et al., 2017) in order to calculate: (i) the percentage of biofilm removal compared to control values after 16 h of treatment; (ii) the minimum biofilm eradicating concentration that removes 50% of the biofilm (MBEC_50_), (iii) the lowest antibiofilm effect (LOABE; lowest concentration needed to observe an antibiofilm effect) and (iv) the specific antibiofilm activity expressed as Δbaseline normalized CI × min^−1^ × mM^−1^. All the experiments were performed in triplicate.

### Safety and Efficacy of the Proteins in Zebrafish

Safety and efficacy of LysRODI and CHAPSH3b were evaluated in two zebrafish models (IKAN Biotech, Navarra, Spain; www.ikanbiotech.com). The experiments and protocols were performed according to the Organization for Economic Cooperation and Development (OECD) following the Standard Guide for Conducting Acute Toxicity Tests (ASTM) and ISO 20776-1:2007 regulations, achieving the Good Laboratory Practices.

Safety was evaluated by the acute toxicity induced in zebrafish embryos by 1 × MIC of the correspondent protein. To do this, eggs were collected after fecundation and kept in a Petri dish containing E3 medium (5 mM NaCl, 0.17 mM KCl, 0.33 mM CaCl_2_, 0.33 mM MgSO_4_, 0.0001% methylene blue) at 34°C until the next day. Zebrafish embryos (*n* = 8) were individually placed in 96-well microplates and 100 μl of the protein was added (0.73 μM for LysRODI and 0.55 μM for CHAPSH3b). As negative and positive controls, 100 μl of E3 medium and 100 μl of E3 supplemented with 1 mg/ml paracetamol (Alfa Aesar, VWR) (Bastiaan Vliegenthart et al., 2014) were used, respectively. The plates were incubated at 34°C and observations were recorded after 72 h. To evaluate the toxicity of the proteins, all embryos were visually inspected (apical parameters, sub-lethal or teratogenic symptoms and mortality). Results are expressed as percentage of surviving embryos. To assess the reproducibility of the model, each experiment (*n* = 8) was performed in triplicate.

To test the lytic efficacy of LysRODI and CHAPSH3b, a systemic model of *S. aureus* infection in adult zebrafish was used. First, zebrafish (*n* = 18) were kept at 34°C for 24 h. Fish were then anesthetized with tricaine MS-222 (Sigma-Aldrich) for 10 min and, intraperitoneally inoculated with 10 μl of a suspension containing ~10^5^ CFU/fish of *S. aureus* ATCC^®^ 25923™, using a Hamilton syringe with 31G gauge needles (BD Microlance). After 1-h post-infection, fish were treated with different concentrations (1 × MIC, 0.5 × MIC or 3 × MIC) of LysRODI or CHAPSH3b, and kept at 34°C for 72 h. The number of surviving zebrafish was recorded for 72 h post-treatment. As control, adult zebrafish (*n* = 18) were infected with the bacteria and inoculated with 0.85% of sodium chloride solution (Sigma Aldrich). The percentage of cumulative survival zebrafish was represented by the Kaplan–Meier analysis.

### Efficacy in a Mouse Model of Mastitis

Male and female CD1 mice of 20–22 g bodyweight were purchased at Charles River International (France) and accommodated in the animal facilities of the “Instituto de Agrobiotecnología” (registration code ES/31-2016-000002-CR-SU-US) with water and food *ad libitum*. Mice handling and procedures were performed in compliance with the current European and International regulations, following the welfare guidelines and recommendations of the Federations of European Laboratories of Animal Science Associations (FELASA) (Rehbinder et al., 2000) and Animal Research Reporting of *in vivo* Experiments (ARRIVE) (Kilkenny et al., 2010) and the study was approved by the Comité de Ética, Experimentación Animal y Bioseguridad (CEEAB) of the Public University of Navarra (PI/018-19).

Pregnancies were synchronized by circadian cycle adjustment and introduction of males in the same cage for 3 days. Ten days after delivery, the offspring were removed and the lactating females (*n* = 7) were treated intramammary. For that, mice were intraperitoneally anesthetized with a mixture of ketamine 100 mg/kg (Imalgene^®^, Merial Laboratorios, S.A) and xylacine 10 mg/kg (Rompun^®^, Bayer Health Care), and, then, treated in the R4 teat by intramammary administration of 0.1 ml containing 24 μg/mouse of LysRODI (*n* = 7) or PBS (*n* = 4) by using a 33 gauge blunt-end needle in a Hamilton syringe (Hamilton). All mice were challenged at 90 min post-treatment, by intramammary inoculation of 5 × 10^4^ CFU/mouse of *S. aureus* Sa10 or *S. epidermidis* B, and, 18 h later, the R4-R5 mammary glands were aseptically removed, individually weighed, serially diluted in PBS (1:9) and plated in agar, in order to determine the number of CFU/gland. An additional control group (*n* = 3) was intramammary inoculated with MOCK buffer and challenged with one of the pathogens (i.e. *S. epidermidis* B) as before. Clinical signs of hyperemia or edema with changes in color, texture or size of the mammary gland were determined by visually inspection after the treatment.

### Statistical Analysis

The SPSS Statistics for Windows V. 22.0 (IBM Corp.) was used for all calculations. The data obtained from the *in vitro* activity tests (specific lytic activity, time-kill assays, biofilm assays and determination of bacterial resistance) were expressed as the mean ± standard deviation of three biological replicates. One-way ANOVA followed by the Student–Newman–Keuls (SNK) or Protected Least Significant Differences (PLSD) post-hoc tests, were used to determine differences in protein activity and also in the mouse model of mastitis. On the other hand, the student t-test was used to compare the differences between the treated and untreated bacterial cultures at a level of significance *p* < 0.05. Statistical comparison of surviving embryos fish was performed by Chi-square test. The percentage of cumulative survival zebrafish was calculated and plotted by Kaplan–Meier cumulative survival analysis and statistically compared by the Log-Rank test, using the StatView^®^ SAS package (SAS Institute Inc., Version 5).

## Results

### New Endolysins LysRODI and LysA72 Were Identified From Phage Genomes

Two new endolysins encoded by bacteriophages infecting *S. aureus* (phiIPLA-RODI and phiIPLA35) were identified (LysRODI and LysA72, respectively) (García et al., 2009), (Gutiérrez et al., 2015). Bioinformatics analysis of these proteins revealed that both possess a modular structure composed by two catalytic domains (CHAP and amidase) and one SH3b cell wall binding domain. LysRODI possesses an amidase belonging to the “type 2” family, while LysA72 carries an amidase “type 3” (Supplementary Figure 1). The predicted 3D structure of the proteins using Phyre2 revealed a modular organization of the domains clearly separated by a linker region (Supplementary Figure 1). LysRODI exhibited a high similarity (>98%) with other endolysins available in public databases, more specifically with those encoded by staphylococcal *Myoviridae* phages (Supplementary Figure 2A). On the other hand, LysA72 has a high similarity with endolysins encoded by other staphylococcal siphophages (Supplementary Figure 2B).

Following computer analysis, the genes coding for LysRODI (54.8 kDa) and LysA72 (53.8 kDa) were cloned into the expression vector pET21a and overexpressed in *E. coli* BL21 (DE3). Nevertheless, after the purification process, proteins LysRODI and LysA72 were not completely soluble, and the concentration of purified protein was 0.6 and 0.3 mg/ml, respectively. To improve the purification efficiency, the endolysin-encoding genes were optimized for expression in *E. coli*. The new constructs resulted in completely soluble proteins with an increased purification yield (2.5 and 1.1 mg/ml, respectively). The chimeric protein CHAPSH3b (30.4 kDa) was purified as described previously with a yield of 0.9 mg/ml (Rodriguez-Rubio et al., 2012).

### LysRODI Shows a High Lytic Activity Against a Broad Range of Staphylococcal Species

The minimum inhibitory concentration (MIC) of the three proteins was calculated against a collection of staphylococcal species including strains from different origins (Table 1). It is worth noting that LysA72 was active only against *S. aureus* and *S. haemolyticus* strains, while LysRODI and CHAPSH3b were able to lyse all the tested staphylococcal species and displayed lower MIC values (Table 1).

Regarding the specific lytic activity (ΔOD_600_ × min^−1^ × μM^−1^), LysRODI and CHAPSH3b showed similar results and were again the most active (*p* < 0.05) (Table 2).

**Table 2:** Specific lytic activity of the proteins against *S. aureus* Sa9 and *S. epidermidis* F12.

	Specific lytic activity	
Protein	*S. aureus* Sa9^a^	*S. epidermidis* F12^a^
LysRODI	0.24 ± 0.02	0.12 ± 0.03
LysA72	0.09 ± 0.00	N.A.
CHAPSH3b	0.20 ± 0.02	0.11 ± 0.02

a *Data are expressed as the mean ± standard deviation of three replicates and expressed as ΔDO_600_ × μM^−1^× min^−1^*.

*N.A.: Not active*.

### Protein Stability and Activity Is Influenced by Different Physicochemical Conditions

**Figure 1:**
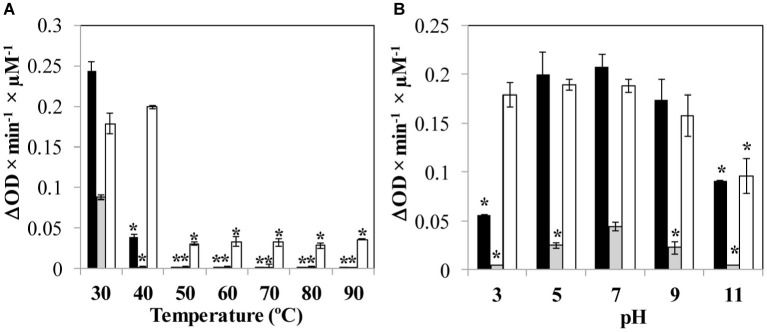
Influence of environmental parameters **(A)** temperature and **(B)** pH on protein activity. Specific lytic activity of the proteins is calculated against *S. aureus* Sa9. Bars represent the activity (ΔDO_600_ × min^−1^ × μM^−1^) of each protein (LysRODI, black; LysA72, gray and CHAPSH3b white). Data are the mean ± standard deviation of three biological replicates. Asterisks indicate statistical differences (*p* < 0.05; Student’s *t*-test) between the specific lytic activity when the protein is submitted to the temperature or pH treatment with the activity observed when the protein is tested at 37°C in NaPi buffer, pH = 7.4. The data were expressed as the mean ± standard deviation of three biological replicates.

The stability of the proteins and the influence of two environmental parameters (temperature and pH) on the lytic activity against *S. aureus* Sa9 was determined. The exposure to temperatures over 40°C completely inactivated LysA72, while LysRODI showed a decreased activity at this temperature and was inactivated at higher temperatures. CHAPSH3b completely retained its activity at 40°C, and even showed some residual activity at higher temperatures (Figure 1A). Regarding pH, the most stable protein was CHAPSH3b, which showed a high activity in a broad range of pH (3–11). In turn, LysRODI and LysA72 showed the highest activity at pH 7, albeit LysRODI was active within pH 5 and 9 (Figure 1B).

Finally, the influence of different ions (KCl, MgCl_2_, NaCl, MnCl_2_, ZnCl_2_, CaCl_2_) on lytic activity was also evaluated. LysRODI was completely inactivated in the presence of MnCl_2_, ZnCl_2_ and MgCl_2_. Interestingly, ZnCl_2_ also resulted inhibitory for LysA72 and CHAPSH3b. Of note, the activity of proteins LysRODI, LysA72 and CHAPSH3b increased ~2-fold by adding NaCl, CaCl_2_ and KCl (data not shown).

### Chimeric Protein LysRODI and CHAPSH3b Lyse Staphylococcal Cells Faster Than LysA72

**Figure 2:**
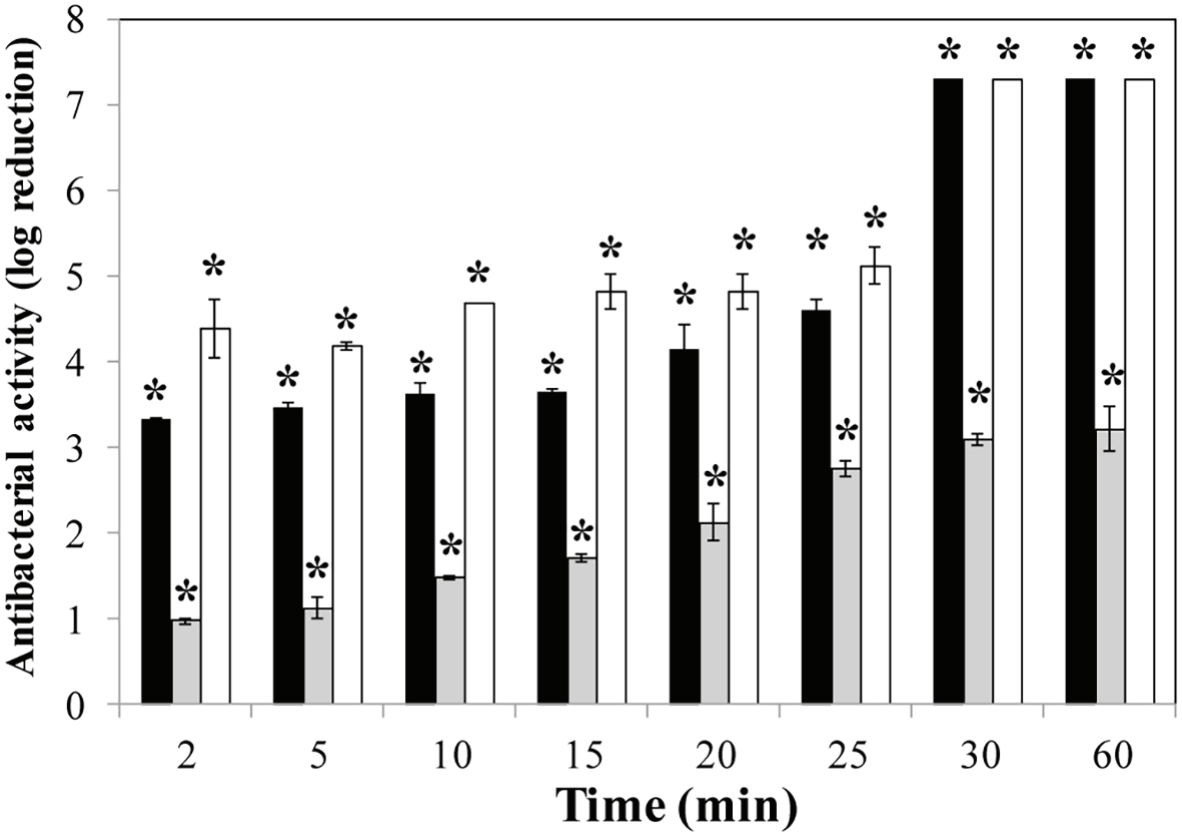
Time-kill curve of *S. aureus* Sa9 treated with equimolar amounts (0.1 μM) of proteins (LysRODI, black; LysA72, gray and CHAPSH3b white). Results (means ± standard deviation of three replicates) are reported as bacterial reduction quantified as the relative inactivation in log units (log_10_[N_0_/N_i_]; N_0_ as the initial number of untreated cells and N_i_ as the number of residual cells counted after treatment). Bars having an asterisk are statistically different (*p* < 0.05) from the untreated control according to the Student’s t-test. The detection limit is 10 CFU/ml.

To determine the killing rate of phage lytic proteins, time-kill curve experiments were performed using *S. aureus* Sa9 cultures (10^7^ CFU/ml) treated with equimolar amounts of each protein (0.1 μM). In these assays, LysRODI and CHAPSH3b showed a higher antibacterial activity than LysA72, as both of them reduced the bacterial counts under the detection limit after 30 min (Figure 2). Of note, CHAPSH3b was faster than the other two proteins since a 4.5 log reduction in viable counts was observed after only 2 min of incubation. At this same time, a 3.5 log reduction was observed for LysRODI. Unfortunately, LysA72 did not achieve a complete elimination of the bacteria (Figure 2).

### Neither the Endolysins nor CHAPSH3b Lead to Staphylococcal Resistance Development

To assess the ability of *S. aureus* and *S. epidermidis* to develop resistance against the phage lytic proteins, we attempted to select resistant mutants in liquid medium by serial subculture of staphylococcal strains in the presence of sub-inhibitory concentrations of the proteins. Lysostaphin was used as a positive control. After 10 rounds of exposure, MIC values were determined and compared with the ones obtained previously for two strains susceptible to the three lytic proteins (Table 3). As expected, the MIC values of lysostaphin increased by more than 32-fold, whereas the studied proteins did not select resistant bacteria.

**Table 3:** MIC values of *S. aureus* Sa9 and *S. epidermidis* F12 before (Control) and after (Treated) exposure to sub-lethal concentrations of different recombinant phage proteins.

Strain	Assay	MIC (μM)^a^			
		LysRODI	LysA72	CHAPSH3b	Lysostaphin
*S. aureus* Sa9	Control	0.57	1.47	0.33	0.43
	Treated^b^	0.49 ± 0.11	1.39 ± 0.24	0.39 ± 0.16	>27.6
*S. epidermidis* LO5081	Control	1.14	2.94 ± 0.09	0.33	1.73
	Treated^b^	1.17 ± 0.06	2.48 ± 0.11	0.32 ± 0.01	>27.6

a *Minimum inhibitory concentration (MIC) expressed as μM*.

b *MIC values after 10 rounds of exposure to protein followed by five additional rounds without protein*.

*Data represent the mean ± standard deviation of three independent biological repeats*.

### LysRODI Shows the Highest and Fastest Antibiofilm Activity *in vitro*

Four different antibiofilm parameters were calculated for the endolysins LysRODI and LysA72 and compared to those previously obtained for CHAPSH3b (Gutierrez et al., 2017) (Table 4). The MBEC_50_ and the LOABE were calculated after treating 8 h preformed biofilms of *S. aureus* 15,981 (a strong biofilm former) with increasing concentrations of the proteins (0.14–9.15 μM for LysRODI and 0.21–13.42 μM for LysA72) (Table 4). LysA72 exhibited the highest MBEC_50_ (minimum biofilm eradication concentration) and LOABE (lowest concentration of protein needed to observe an antibiofilm effect) values indicating that a higher concentration is necessary to attain the same antibiofilm effect. In turn, LysRODI possessed the lowest MBEC_50_ value of all three proteins, since only 2.21 μM could remove 50% of the biofilm.

**Table 4:** Antibiofilm activity of LysRODI, LysA72 and CHAPSH3b.

Protein	MBEC_50_ (μM)^a^	LOABE (μM)^b^	Specific antibiofilm activity^c^	Biofilm reduction (%)^d^
LysRODI	2.21 ± 0.22	1.14	4.53 ± 0.63	94.23 ± 4.59
LysA72	13.26 ± 3.87	13.42	0.12 ± 0.02	17.13 ± 2.63
CHAPSH3b^e^	4.4 ± 1.2	1.2	0.38 ± 0.03	25.62 ± 6.23

a *MBEC_50_: minimum biofilm eradicating concentration that removes 50% of the biofilm*.

b *LOABE: lowest antibiofilm effect. MBEC_50_ and LOABE were calculated for S. aureus 15,981 8 h mature biofilms treated with increasing concentrations of protein*.

c *Specific antibiofilm activity expresses as the mean ± standard deviation of (Δbaseline normalized CI × mM^−1^ × min^−1^) using 7 μM of protein*.

d *% of biofilm reduction expresses as the mean ± standard deviation (n = 3) of normalized CI using 7 μM of protein at 16 h post-treatment and 37°C*.

e *These data have been previously published (22). All data represent mean ± standard deviation of three biological replicates*.

In order to compare the specific antibiofilm activity of the proteins, an equimolar amount of protein (7 μM) was used to treat the biofilms (Table 4). Interestingly, LysRODI showed the highest specific antibiofilm activity (~4.5), being 11 and 38 times more active than CHAPSH3b and LysA72, respectively. This result indicates a faster antibiofilm activity of LysRODI, since maximum biofilm removal was achieved 1 h after treatment, while for CHAPSH3b and LysA72 the maximum reduction was achieved after 2.5 and 5 h, respectively (data not shown). Moreover, the percentage of biofilm reduction after 16 h of treatment with LysRODI was 94% but only 25 and 17% when treated with CHAPSH3b and LysA72, respectively (Table 4).

### Both LysRODI and CHAPSH3b Are Safe and Show Anti-Staphylococcal Activity in Zebrafish

**Figure 3:**
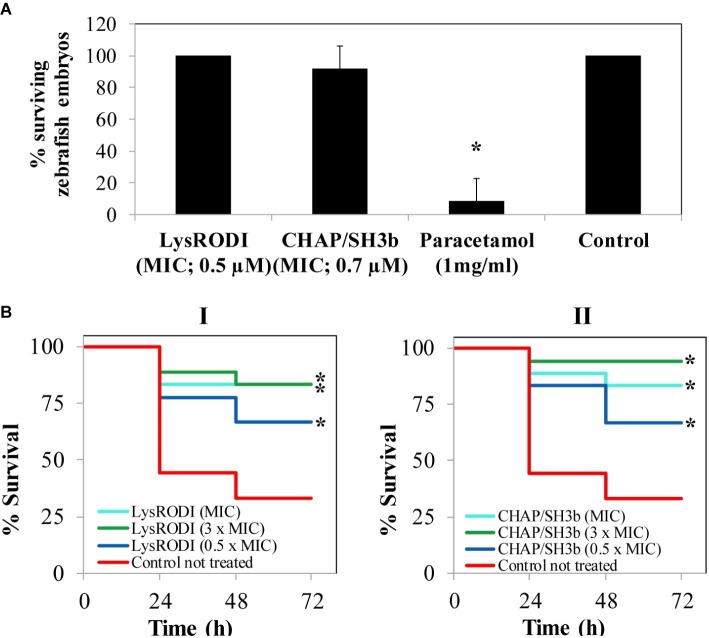
Evaluation of safety and activity of LysRODI and CHAPSH3b using a zebrafish model. **(A)** Acute toxicity of the proteins was tested by exposure of zebrafish embryos (*n* = 8) to 1 × MIC of LysRODI or CHAPSH3b. Medium E3 was used as a negative control and 1 mg/ml paracetamol, as a positive control. Bars represent mean ± standard deviation (*n* = 24) of triplicate experiments. The asterisk indicates statistical differences (*p* < 0.05) *vs* untreated control by Chi-square test. **(B)** Efficacy of LysRODI (I) and CHAPSH3b (II) in a zebrafish model infected with ~10^5^ CFU/fish of *S. aureus* when treating with 0.5 × MIC, 1 × MIC and 3 × MIC. Kaplan–Meier graphs represent the percentage of cumulative survival zebrafish (*n* = 18) observed at 24, 48 and 72 h. Asterisks indicate significant differences (*p* < 0.05) *vs.* the untreated control according to the Kaplan–Meier cumulative survival plot and statistically compared by the Log-Rank test.

Based on the *in vitro* activity data, LysRODI and CHAPSH3b were assessed *in vivo* in a zebrafish model. Initially, acute toxicity was evaluated by exposure of zebrafish embryos to 1 × MIC of LysRODI (0.5 μM) and CHAPSH3b (0.7 μM). As expected, treatment with 1 mg/ml of paracetamol was toxic for the embryos (Figure 3A). In protein-treated groups embryos survived in presence of LysRODI and CHAPSH3b (>92% survival), indicating a non-toxic effect (Figure 3A).

Once established the lack of toxicity of the lytic proteins, their protective effect against a subsequent bacterial challenge was evaluated in a systemic model of infection. As expected, the untreated zebrafish control infected with *S. aureus* showed a 33.3% of cumulative survival after 72 h (Figure 3B). In contrast, treatment with three different doses (0.5 × MIC, 1 × MIC or 3 × MIC) of LysRODI (0.25, 0.5 and 1 μM) or CHAPSH3b (0.35, 0.7 and 1.4 μM) significantly improved survival of the infected animals (*p* = 0.0028 and *p* = 0.0002, respectively), with similar levels of efficacy obtained for both proteins testing 3 different concentrations (*p* > 0.05; Supplementary Table 1). In fact, at 72 h after treatment, MIC and 0.5 × MIC of both proteins allowed a 66.7 and 83.3% survival, respectively, although these differences were not statistically significant (*p* < 0.05). Of note, 3 × MIC of LysRODI led to a 77.7% survival, which is not statistically significant compared to the other two concentrations tested (*p* < 0.05). Meanwhile, 3 × MIC of CHAPSH3b led to a 94.4% of survival which is statistically significant (*p* > 0.05) when compared with 0.5 × MIC, indicating a dose-dependent effect. Besides, the treatment with 3 × MIC of CHAPSH3b or LysRODI gave similar results (*p* < 0.05) Moreover, no viable bacteria could be detected in any of the surviving fish 72 h post infection (data not shown).

### LysRODI Prevents Staphylococcal Infection in a Mouse Model of Mastitis

**Figure 4:**
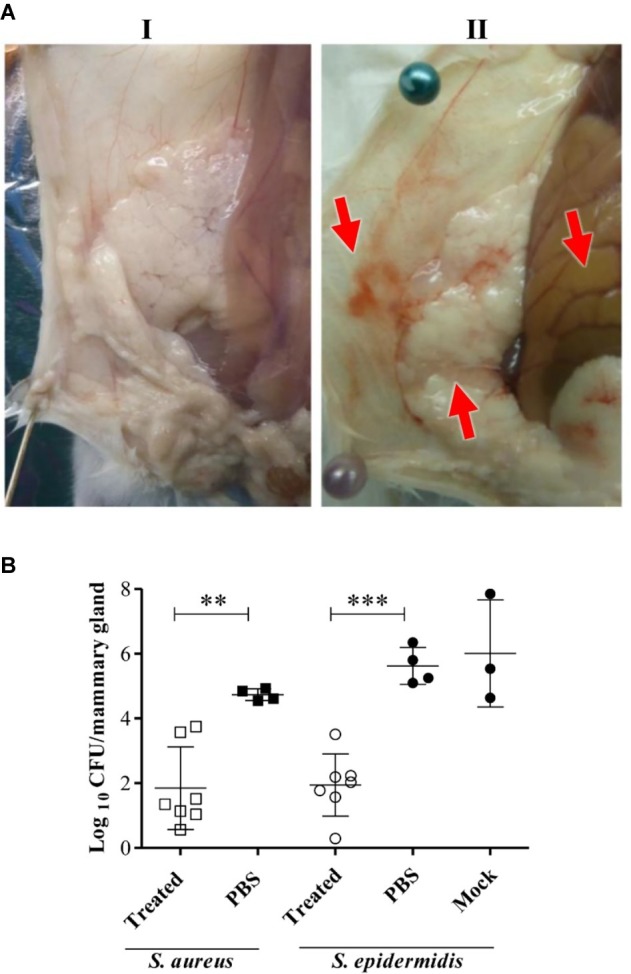
Efficacy of LysRODI preventive treatment in a mastitis mouse model. CD1 lactating mice at breastfeeding day 10 were treated by intramammary administration of 24 μg of LysRODI and challenged 90 min later with 5 × 10^4^ CFU/mice of *S. epidermidis* or *S. aureus.* Mice inoculated with PBS or mock buffer were used as controls of staphylococcal infection. **(A)** Macroscopic aspect of R4-R5 mammary glands of mice treated with (I) treated 24 μg/mouse of LysRODI or (II) PBS and infected with *S. aureus* Sa10. Red arrows indicate symptoms of edema, hyperemia and changes in color of an infected mammary gland. **(B)** Preventive efficacy of LysRODI against *S. aureus* and *S. epidermidis* mammary glands infections in CD1 mice, determined by the mean ± standard deviation of individual log_10_ CFU/mammary gland. White symbols are Lys-RODI treated mice; black symbols are untreated mice, inoculated with either PBS or mock sterile buffers; circles and squares represent, respectively, *S. aureus* and *S. epidermidis* challenged mice. The number of CFU was determined in each R4-R5 mammary gland, at 18 h post-challenge. Statistical comparison of means was performed by ANOVA and post-hoc PLSD tests: ^**^p < 0.001 and ^***^p < 0.0001 vs. control (PBS or mock).

LysRODI was selected to evaluate its *in vivo* ability to prevent *S. aureus* and *S. epidermidis* mastitis in a murine model, since it showed the most effective antibiofilm activity and its *in vivo* activity was dose-independent in a zebrafish infection model. Intramammary treatment with LysRODI in mice prevented the mastitis lesions (Figure 4A) and significantly reduced the bacterial virulent infection caused by *S. aureus* (*p* < 0.001) or *S. epidermidis* (*p* < 0.0001) (Figure 4B). Indeed, mice treated with the endolysin showed healthy mammary glands in terms of color, texture, size and absence of edema or hyperemia, in contrast to untreated controls (Figure 4A). Regarding the bacterial burden, LysRODI treated mice showed a reduction of 3–4 log units compared to the untreated mice controls (Figure 4B).

## Discussion

Phage lytic proteins could potentially reduce the incidence of animal diseases while avoiding antibiotic use. In the case of *S. aureus* and *S. epidermidis* infections, prevention is perhaps a better strategy than treatment. For instance, once a staphylococcal infection is established, the bacterial cells can be found in an intracellular stage or forming antibiotic-resistant biofilms. Previous studies have shown that phage lytic proteins can be effective as a preventive strategy against staphylococcal infections by decolonization of skin and nares in murine models (Fenton et al., 2010; Pastagia et al., 2011; Paul et al., 2011).

The activity and stability of three proteins LysRODI, LysA72 and CHAPSH3b was firstly evaluated *in vitro* against staphylococcal strains isolated from human and farm animals. Activity was assessed by muralytic, MIC and time-kill assays (Schmelcher et al., 2012a). The specific lytic activity of LysRODI was similar to that of the well-known endolysin LysK (~0.04 ΔDO_600_ × min^−1^ × μg^−1^) (Becker et al., 2008), with which it shares 98% of protein similarity. Interestingly, LysRODI and CHAPSH3b turned out to be active against all the species of staphylococci tested. This broad activity range is quite common among staphylococcal endolysins since their cell wall binding domain (CBD) have proven to recognize different staphylococcal species (Becker et al., 2009; Schmelcher et al., 2015b; Gutierrez et al., 2018). In addition to the CBD, the broad activity range can also be explained by the cleavage sites of the CHAP and amidase domains of staphylococcal endolysins, which are conserved in the peptidoglycan of both *S. aureus* and other staphylococcal species. Surprisingly, the activity of LysA72 seems to be restricted to *S. aureus* and *S. haemolyticus* strains. Unfortunately, it is difficult to determine if this narrow range is related to the protein structure because there is only one protein with a high similarity (98%) to LysA72 that has been purified to date; endolysins from phage phi12. The activity of this endolysin could not be determined due to cell aggregation during the turbidity assay, which led to an increase in optical density (Sass and Bierbaum, 2007). This phenomenon of cell aggregation was explained by the authors because of an aminoacidic exchange in position 260 (Amidase-3 domain), which is only present in the endolysin from phage phi12 but not in LysA72 or other related endolysins (Becker et al., 2009).

Protein stability under a wide range of environmental conditions is very important for the development of endolysin-based antimicrobial products, especially those aimed for topical application. Both CHAPSH3b and LysRODI retained full activity for at least 30 min in skin-simulated conditions (pH between 4 and 7, temperature from 26 to 38°C) (Lambers et al., 2006). The activity of LysRODI under physicochemical disadvantages are in accordance with those previously obtained for LysK, both active within the same pH range (5–9), temperature (stable until 40°C) and are positively influenced by the addition of NaCl (Becker et al., 2008).

Time-killing assays provide a good indication of the speed at which a given antimicrobial can kill bacterial cells, which in turn is indicative of its effectiveness to prevent infection progress. In this study, a similar time-killing speed was observed for LysRODI and CHAPSH3b, whereas LysA72 was slower and never caught up with the other two proteins in terms of total bacterial elimination. Generally, the quicker activity of endolysins is consistent with previous reports due to the high affinity of CBDs to the bacterial peptidoglycan (Liu et al., 2015). Of note, the ability of LysRODI and CHAPSH3b to remove 7 log units of bacteria is even higher than that of CF-301, a lysin encoded within a prophage of the *Streptococcus suis* genome, whose domain arrangement is very similar to that of CHAPSH3b (CHAP domain and SH3b domain). In fact, addition of CF-301at its minimum inhibitory concentration only led to a reduction in bacterial cells of about 3 log units within 30 min (Schuch et al., 2014).

Attachment to host tissues and biofilm formation are processes of key importance for persistence and progress of bacterial infections (Otto, 2013). In terms of preventive treatment, any successful strategy should hinder this process and display antibiofilm activity. Removal of preformed biofilms, however, is expected to be hindered by their maturation state and their complex three-dimensional structure, which limits access of antimicrobials to their target bacteria. Nonetheless, there are promising results with phage lytic proteins. In this study, LysRODI displayed the best antibiofilm activity on the basis of different antibiofilm parameters. Generally speaking, it has been demonstrated that complete removal of a mature biofilm (more than 24 h old biofilms) needs a second endolysin treatment (Gutiérrez et al., 2014) or a combined treatment with antibiotics (Chopra et al., 2015). Indeed, although CF-301 showed a potent antibiofilm activity (MBEC_90_ values from 9 nM to 0.3 μM), it was improved when combining with lysostaphin (Schuch et al., 2017).

We also evaluated the *in vivo* activity of LysRODI and CHAPSH3b, by using a zebra fish model, which has been deemed suitable for assessing the efficacy of antimicrobial agents before proceeding to mammalian studies (Díez-Martínez et al., 2013). Initially, we observed that none of the proteins was toxic for zebrafish embryos at the dose tested. In a subsequent experiment, a single dose of either protein significantly reduced mortality of zebrafish by *S. aureus* in a systemic model of infection. Nevertheless, the treatment with CHAPSH3b seems to be dose dependent. Due to the positive results obtained in the zebrafish model, we decided to assess the efficacy of the protein that resulted in the best overall results (LysRODI) in a murine mastitis model. Even though the effectiveness of endolysins has been demonstrated *in vivo*, there is a lack of research regarding their safety and toxicity. Nevertheless, safety studies carried out with endolysins Cpl-1 and PaI supported the safety of these compounds, since there was no significant pro-inflammatory response, hypersensitivity or allergic reaction after injecting 0.3 mg per mouse (15 mg/kg) of each protein (Harhala et al., 2018). Moreover, toxicity in mammals is not a worrying issue since it has been proven that intravenous administration of endolysin SAL-200 (highly similar to LysRODI) showed no signs of toxicity in rodents after single- and repeated-dose experiments (Jun et al., 2014). Moreover, SAL-200 was also administered (single intravenous) among healthy volunteers, with no adverse side-effects reported (Jun et al., 2017).

The effectiveness of endolysins against staphylococcal infections in animal models has been widely demonstrated (Gutierrez et al., 2018). Specifically, for mastitis treatment, two promising papers support the use of phage lytic proteins as therapeutic agents. Schmelcher et al. (2012b) demonstrated that the infusion of a combination of proteins (ƛSA2-E-LysK-SH3b and lysostaphin, 12.5 μg each/gland) caused a 3.36-log decrease in viable bacteria and a reduction in inflammation indicators in a murine mastitis. More recently, preliminary results showed that endolysin Trx-SA1 (20 mg once per day) could effectively control mild clinical mastitis caused by *S. aureus* in cows (Fan et al., 2016). Nonetheless, an endolysin-based treatment could be challenging, as bacterial cells could form biofilms on host tissues or live intracellularly. For this reason, it would be preferable to perform preventive rather than therapeutic treatments. However, few reports have been published about prophylaxis of infectious diseases caused by *S. aureus* using phage lytic proteins and none regarding *S. epidermidis*. Indeed, the emergence of *S. aureus* resistance to mupirocin has led clinicians to rethink its use for nasal decolonization in certain groups of patients (Coates et al., 2009). In contrast, phage lytic proteins like ClyS have shown a greater potential than mupirocin for topical eradication of this bacterium and a lower potential for resistance development (Pastagia et al., 2011). Here, we proved that intrammamary infusion of 24 μg (0.43 μM) of LysRODI can protect mice from *S. aureus* and *S. epidermidis* mastitis. It is also worth noting that no resistant bacteria were obtained after sub-lethal exposure to the proteins. Previous studies found similar results with other endolysins (Pastagia et al., 2011; Gutierrez et al., 2018), with the exception of LysK, which led to a 42-fold MIC increase in *S. aureus* liquid cultures (Becker et al., 2016). Nonetheless, in the case of bovine mastitis, the use of endolysins as prophylaxis (every animal is treated on a regular basis by intramammary infusion) or as treatment needs to be evaluated in detail, due to the expected high cost derived from production of these enzymes.

Overall, phage lytic proteins offer ideal features, including their easy manipulation for designing new proteins with improved activity and the lack of bacterial resistance development, to be used as therapeutic agents and as decolonizing products for veterinary applications.

## Data Availability Statement

All datasets generated for this study are included in the article/Supplementary Material.

## Ethics Statement

The animal study was reviewed and approved by registration code ES/31-2016-000002-CR-SU-US.

## Author Contributions

PG, AR, and MG conceived and designed the experiments. DG, LF, and SP performed the experiments related with the *in vitro* characterization of the proteins. VG performed the murine model experiments. DG and VG analyzed the data. All the authors wrote the paper.

### Conflict of Interest

The authors declare that the research was conducted in the absence of any commercial or financial relationships that could be construed as a potential conflict of interest.

## Footnotes

^1^https://www.who.int/news-room/fact-sheets/detail/antimicrobial-resistance

^2^http://www.who.int/medicines/publications/global-priority-list-antibiotic-resistant-bacteria/en/

^3^hmmer.org

^4^https://www.geneious.com

^5^https://www.genscript.com/codon-opt.html

## References

[ref1] Altschul S. F. Gish W. Miller W. Myers E. W. Lipman D. J. (1990). Basic local alignment search tool. J. Mol. Biol. 215, 403–410. 10.1016/S0022-2836(05)80360-2 2231712

[ref500] Bastiaan Vliegenthart A. D. Tucker C. S. Del Pozo J. Dear J. W. (2014). Zebrafish as model organisms for studying drug-induced liver injury. Br. J. Clin. Pharmacol. 78, 1217–1227. 10.1111/bcp.12408, PMID: 24773296 PMC4219854

[ref2] Becker S. C. Foster-Frey J. Donovan D. M. (2008). The phage K lytic enzyme LysK and lysostaphin act synergistically to kill MRSA. FEMS Microbiol. Lett. 287, 185–191. 10.1111/j.1574-6968.2008.01308.x, PMID: 18721148

[ref3] Becker S. C. Foster-Frey J. Stodola A. J. Anacker D. Donovan D. M. (2009). Differentially conserved staphylococcal SH3b_5 cell wall binding domains confer increased staphylolytic and streptolytic activity to a streptococcal prophage endolysin domain. Gene 443, 32–41. 10.1016/j.gene.2009.04.023, PMID: 19422893

[ref4] Becker S. C. Roach D. R. Chauhan V. S. Shen Y. Foster-Frey J. Powell A. M.. (2016). Triple-acting lytic enzyme treatment of drug-resistant and intracellular *Staphylococcus aureus*. Sci. Rep. 6:25063. 10.1038/srep25063, PMID: 27121552 PMC4848530

[ref5] Blazquez B. Fresco-Taboada A. Iglesias-Bexiga M. Menendez M. Garcia P. (2016). PL3 amidase, a tailor-made lysin constructed by domain shuffling with potent killing activity against pneumococci and related species. Front. Microbiol. 7:1156. 10.3389/fmicb.2016.01156, PMID: 27516758 PMC4963390

[ref6] CLSI (2015). Performance standards for antimicrobial susceptibility testing; twenty-fifth informational supplement. CLSI approved document M100-S25. Wayne, PA: Clinical and Laboratory Standards Institute.

[ref7] Coates T. Bax R. Coates A. (2009). Nasal decolonization of *Staphylococcus aureus* with mupirocin: strengths, weaknesses and future prospects. J. Antimicrob. Chemother. 64, 9–15. 10.1093/jac/dkp159, PMID: 19451132 PMC2692503

[ref8] Czaplewski L. Bax R. Clokie M. Dawson M. Fairhead H. Fischetti V. A.. (2016). Alternatives to antibiotics-a pipeline portfolio review. Lancet Infect. Dis. 16, 239–251. 10.1016/S1473-3099(15)00466-1, PMID: 26795692

[ref9] Chang Y. Yoon H. Kang D. H. Chang P. S. Ryu S. (2017). Endolysin LysSA97 is synergistic with carvacrol in controlling *Staphylococcus aureus* in foods. Int. J. Food Microbiol. 244, 19–26. 10.1016/j.ijfoodmicro.2016.12.007, PMID: 28063330

[ref10] Chopra S. Harjai K. Chhibber S. (2015). Potential of sequential treatment with minocycline and *S. aureus* specific phage lysin in eradication of MRSA biofilms: an *in vitro* study. Appl. Microbiol. Biotechnol. 99, 3201–3210. 10.1007/s00253-015-6460-1, PMID: 25707865

[ref11] Delgado S. Arroyo R. Jiménez E. Marín M. L. del Campo R. Fernández L.. (2009). *Staphylococcus epidermidis* strains isolated from breast milk of women suffering infectious mastitis: potential virulence traits and resistance to antibiotics. BMC Microbiol. 9:82. 10.1186/1471-2180-9-82, PMID: 19422689 PMC2685400

[ref12] Díez-Martínez R. de Paz H. D. Bustamante N. García E. Menéndez M. García P. (2013). Improving the lethal effect of cpl-7, a pneumococcal phage lysozyme with broad bactericidal activity, by inverting the net charge of its cell wall-binding module. Antimicrob. Agents Chemother. 57, 5355–5365. 10.1128/AAC.01372-13, PMID: 23959317 PMC3811316

[ref13] Donovan D. M. Lardeo M. Foster-Frey J. (2006). Lysis of staphylococcal mastitis pathogens by bacteriophage phi11 endolysin. FEMS Microbiol. Lett. 265, 133–139. 10.1111/j.1574-6968.2006.00483.x, PMID: 17054440

[ref14] European Commission (2017). A European one health action plan against antimicrobial resistance AMR. Action Plan European Commission. Brussels: European Commission, 1–24.

[ref15] Fan J. Zeng Z. Mai K. Yang Y. Feng J. Bai Y.. (2016). Preliminary treatment of bovine mastitis caused by *Staphylococcus aureus*, with trx-SA1, recombinant endolysin of *S. aureus* bacteriophage IME-SA1. Vet. Microbiol. 191, 65–71. 10.1016/j.vetmic.2016.06.001, PMID: 27374909

[ref16] Fenton M. Casey P. G. Hill C. Gahan C. G. Ross R. P. McAuliffe O. (2010). The truncated phage lysin CHAP(k) eliminates *Staphylococcus aureus* in the nares of mice. Bioeng. Bugs 1, 404–407. 10.4161/bbug.1.6.13422 21468207 PMC3056090

[ref17] Fernandez L. Gonzalez S. Campelo A. B. Martinez B. Rodriguez A. Garcia P. (2017). Downregulation of autolysin-encoding genes by phage-derived lytic proteins inhibits biofilm formation in *Staphylococcus aureus*. Antimicrob. Agents Chemother. 61:e02724-16. 10.1128/AAC.02724-16, PMID: 28289031 PMC5404533

[ref18] Fischetti V. A. (2010). Bacteriophage endolysins: a novel anti-infective to control gram-positive pathogens. Int. J. Med. Microbiol. 300, 357–362. 10.1016/j.ijmm.2010.04.002, PMID: 20452280 PMC3666336

[ref19] García P. Martínez B. Obeso J. M. Lavigne R. Lurz R. Rodríguez A. (2009). Functional genomic analysis of two *Staphylococcus aureus* phages isolated from the dairy environment. Appl. Environ. Microbiol. 75, 7663–7673. 10.1128/AEM.01864-09, PMID: 19837832 PMC2794103

[ref20] García P. Martínez B. Rodríguez L. Rodríguez A. (2010). Synergy between the phage endolysin LysH5 and nisin to kill *Staphylococcus aureus* in pasteurized milk. Int. J. Food Microbiol. 141, 151–155. 10.1016/j.ijfoodmicro.2010.04.029, PMID: 20537744

[ref21] Gomes F. Saavedra M. J. Henriques M. (2016). Bovine mastitis disease/pathogenicity: evidence of the potential role of microbial biofilms. Pathog. Dis. 74:ftw006. 10.1093/femspd/ftw006, PMID: 26772653

[ref22] Gutierrez D. Briers Y. Rodriguez-Rubio L. Martinez B. Rodriguez A. Lavigne R.. (2015). Role of the pre-neck appendage protein (Dpo7) from phage vB_SepiS-phiIPLA7 as an anti-biofilm agent in staphylococcal species. Front. Microbiol. 6:1315. 10.3389/fmicb.2015.01315, PMID: 26635776 PMC4658415

[ref23] Gutierrez D. Fernandez L. Martinez B. Ruas-Madiedo P. Garcia P. Rodriguez A. (2017). Real-time assessment of *Staphylococcus aureus* biofilm disruption by phage-derived proteins. Front. Microbiol. 8:1632. 10.3389/fmicb.2017.01632, PMID: 28883818 PMC5573737

[ref24] Gutierrez D. Fernandez L. Rodriguez A. Garcia P. (2018). Are phage lytic proteins the secret weapon to kill *Staphylococcus aureus*? MBio 9:e01923-17. 10.1128/mBio.01923-17, PMID: 29362234 PMC5784253

[ref25] Gutiérrez D. Hidalgo-Cantabrana C. Rodríguez A. García P. Ruas-Madiedo P. (2016). Monitoring in real time the formation and removal of biofilms from clinical related pathogens using an impedance-based technology. PLoS One 11:e0163966. 10.1371/journal.pone.0163966, PMID: 27695058 PMC5047529

[ref26] Gutierrez D. Rodriguez-Rubio L. Martinez B. Rodriguez A. Garcia P. (2016). Bacteriophages as weapons against bacterial biofilms in the food industry. Front. Microbiol. 7:825. 10.3389/fmicb.2016.00825, PMID: 27375566 PMC4897796

[ref27] Gutiérrez D. Ruas-Madiedo P. Martínez B. Rodríguez A. García P. (2014). Effective removal of staphylococcal biofilms by the endolysin LysH5. PLoS One 9:e107307. 10.1371/journal.pone.0107307, PMID: 25203125 PMC4159335

[ref28] Gutiérrez D. Vandenheuvel D. Martínez B. Rodríguez A. Lavigne R. García P. (2015). Two phages, phiIPLA-RODI and phiIPLA-C1C, lyse mono- and dual-species staphylococcal biofilms. Appl. Environ. Microbiol. 81, 3336–3348. 10.1128/AEM.03560-14, PMID: 25746992 PMC4407228

[ref29] Harhala M. Nelson D. C. Miernikiewicz P. Heselpoth R. D. Brzezicka B. Majewska J.. (2018). Safety studies of pneumococcal endolysins Cpl-1 and Pal. Viruses 10:E638. 10.3390/v10110638, PMID: 30445722 PMC6266847

[ref30] Hogeveen H. Huijps K. Lam T. J. (2011). Economic aspects of mastitis: new developments. New Zealand Vet. J. 59, 16–23. 10.1080/00480169.2011.547165, PMID: 21328153

[ref31] Jun S. Y. Jang I. J. Yoon S. Jang K. Yu K. S. Cho J. Y.. (2017). Pharmacokinetics and tolerance of the phage endolysin-based candidate drug SAL200 after a single intravenous administration among healthy volunteers. Antimicrob. Agents Chemother. 61:e02629-16. 10.1128/AAC.02629-16, PMID: 28348152 PMC5444177

[ref32] Jun S. Y. Jung G. M. Yoon S. J. Choi Y. J. Koh W. S. Moon K. S.. (2014). Preclinical safety evaluation of intravenously administered SAL200 containing the recombinant phage endolysin SAL-1 as a pharmaceutical ingredient. Antimicrob. Agents Chemother. 58, 2084–2088. 10.1128/AAC.02232-13, PMID: 24449776 PMC4023757

[ref33] Kelley L. A. Mezulis S. Yates C. M. Wass M. N. Sternberg M. J. (2015). The Phyre2 web portal for protein modeling, prediction and analysis. Nat. Protoc. 10, 845–858. 10.1038/nprot.2015.053, PMID: 25950237 PMC5298202

[ref34] Kilkenny C. Browne W. J. Cuthill I. C. Emerson M. Altman D. G. (2010). Improving bioscience research reporting: the ARRIVE guidelines for reporting animal research. PLoS Biol. 8:e1000412. 10.1371/journal.pbio.1000412, PMID: 20613859 PMC2893951

[ref35] Lambers H. Piessens S. Bloem A. Pronk H. Finkel P. (2006). Natural skin surface pH is on average below 5, which is beneficial for its resident flora. Int. J. Cosmet. Sci. 28, 359–370. 10.1111/j.1467-2494.2006.00344.x, PMID: 18489300

[ref36] Liu J. Zhang X. Yang H. Yuan J. Wei H. Yu J.. (2015). Study of the interactions between endolysin and bacterial peptidoglycan on *S. aureus* by dynamic force spectroscopy. Nanoscale 7, 15245–15250. 10.1039/C5NR03525B, PMID: 26324763

[ref37] Martín V. Maldonado-Barragán A. Moles L. Rodríguez-Banos M. Campo R. D. Fernández L.. (2012). Sharing of bacterial strains between breast milk and infant feces. J. Hum. Lact. 28, 36–44. 10.1177/0890334411424729, PMID: 22267318

[ref38] Nyman A. K. Fasth C. Waller K. P. (2018). Intramammary infections with different non-aureus staphylococci in dairy cows. J. Dairy Sci. 101, 1403–1418. 10.3168/jds.2017-13467, PMID: 29174143

[ref39] Obeso J. M. Martinez B. Rodriguez A. Garcia P. (2008). Lytic activity of the recombinant staphylococcal bacteriophage PhiH5 endolysin active against *Staphylococcus aureus* in milk. Int. J. Food Microbiol. 128, 212–218. 10.1016/j.ijfoodmicro.2008.08.010 18809219

[ref40] Oliveira H. Melo L. D. Santos S. B. Nobrega F. L. Ferreira E. C. Cerca N.. (2013). Molecular aspects and comparative genomics of bacteriophage endolysins. J. Virol. 87, 4558–4570. 10.1128/JVI.03277-12, PMID: 23408602 PMC3624390

[ref41] Otto M. (2013). Staphylococcal infections: mechanisms of biofilm maturation and detachment as critical determinants of pathogenicity. Annu. Rev. Med. 64, 175–188. 10.1146/annurev-med-042711-140023, PMID: 22906361

[ref42] Pastagia M. Euler C. Chahales P. Fuentes-Duculan J. Krueger J. G. Fischetti V. A. (2011). A novel chimeric lysin shows superiority to mupirocin for skin decolonization of methicillin-resistant and -sensitive *Staphylococcus aureus* strains. Antimicrob. Agents Chemother. 55, 738–744. 10.1128/AAC.00890-10, PMID: 21098252 PMC3028755

[ref43] Paul V. D. Rajagopalan S. S. Sundarrajan S. George S. E. Asrani J. Y. Pillai R.. (2011). A novel bacteriophage tail-associated muralytic enzyme (TAME) from phage K and its development into a potent antistaphylococcal protein. BMC Microbiol. 11:226. 10.1186/1471-2180-11-226, PMID: 21985151 PMC3207973

[ref44] Rehbinder C. Alenius S. Bures J. de las Heras M. L. Greko C. Kroon P. S. (2000). FELASA recommendations for the health monitoring of experimental units of calves, sheep and goats report of the federation of European Laboratory Animal Science Associations (FELASA) working group on animal health. Lab. Anim. 34, 329–350. 10.1258/002367700780387723 11072854

[ref45] Reichelt P. Schwarz C. Donzeau M. (2006). Single step protocol to purify recombinant proteins with low endotoxin contents. Protein Expr. Purif. 46, 483–488. 10.1016/j.pep.2005.09.027, PMID: 16290005

[ref46] Rodriguez-Rubio L. Gutierrez D. Donovan D. M. Martinez B. Rodriguez A. Garcia P. (2016a). Phage lytic proteins: biotechnological applications beyond clinical antimicrobials. Crit. Rev. Biotechnol. 36, 542–552. 10.3109/07388551.2014.993587 25603721

[ref47] Rodriguez-Rubio L. Chang W. L. Gutierrez D. Lavigne R. Martinez B. Rodriguez A. (2016b). ‘Artilysation’ of endolysin lambdaSa2lys strongly improves its enzymatic and antibacterial activity against streptococci. Sci. Rep. 6:35382. 10.1038/srep35382 27775093 PMC5075790

[ref48] Rodriguez-Rubio L. Martinez B. Rodriguez A. Donovan D. M. Garcia P. (2012). Enhanced staphylolytic activity of the *Staphylococcus aureus* bacteriophage vB_SauS-phiIPLA88 HydH5 virion-associated peptidoglycan hydrolase: fusions, deletions, and synergy with LysH5. Appl. Environ. Microbiol. 78, 2241–2248. 10.1128/AEM.07621-11, PMID: 22267667 PMC3302612

[ref49] Rodríguez L. Martínez B. Zhou Y. Rodríguez A. Donovan D. M. García P. (2011). Lytic activity of the virion-associated peptidoglycan hydrolase HydH5 of *Staphylococcus aureus* bacteriophage vB_SauS-phiIPLA88. BMC Microbiol. 11, 138. 10.1186/1471-2180-11-138, PMID: 21682850 PMC3150257

[ref50] Sass P. Bierbaum G. (2007). Lytic activity of recombinant bacteriophage phi11 and phi12 endolysins on whole cells and biofilms of *Staphylococcus aureus*. Appl. Environ. Microbiol. 73, 347–352. 10.1128/AEM.01616-06 17085695 PMC1797112

[ref51] Schmelcher M. Donovan D. M. Loessner M. J. (2012a). Bacteriophage endolysins as novel antimicrobials. Future Microbiol. 7, 1147–1171. 10.2217/fmb.12.97 23030422 PMC3563964

[ref52] Schmelcher M. Powell A. M. Becker S. C. Camp M. J. Donovan D. M. (2012b). Chimeric phage lysins act synergistically with lysostaphin to kill mastitis-causing *Staphylococcus aureus* in murine mammary glands. Appl. Environ. Microbiol. 78, 2297–2305. 10.1128/AEM.07050-11 22286996 PMC3302589

[ref53] Schmelcher M. Powell A. M. Camp M. J. Pohl C. S. Donovan D. M. (2015a). Synergistic streptococcal phage lambdaSA2 and B30 endolysins kill streptococci in cow milk and in a mouse model of mastitis. Appl. Microbiol. Biotechnol. 99, 8475–8486. 10.1007/s00253-015-6579-0 25895090 PMC4573782

[ref54] Schmelcher M. Shen Y. Nelson D. C. Eugster M. R. Eichenseher F. Hanke D. C. (2015b). Evolutionarily distinct bacteriophage endolysins featuring conserved peptidoglycan cleavage sites protect mice from MRSA infection. J. Antimicrob. Chemother. 70, 1453–1465. 10.1093/jac/dku552 25630640 PMC4398471

[ref55] Schuch R. Lee H. M. Schneider B. C. Sauve K. L. Law C. Khan B. K.. (2014). Combination therapy with lysin CF-301 and antibiotic is superior to antibiotic alone for treating methicillin-resistant *Staphylococcus aureus*-induced murine bacteremia. J. Infect. Dis. 209, 1469–1478. 10.1093/infdis/jit637, PMID: 24286983 PMC3982849

[ref56] Schuch R. Khan B. K. Raz A. Rotolo J. A. Wittekind M. (2017). Bacteriophage yysin CF-301, a potent antistaphylococcal biofilm agent. Antimicrob. Agents Chemother. 61:e02666-16. 10.1128/AAC.02666-16, PMID: 28461319 PMC5487678

[ref57] Valle J. Toledo-Arana A. Berasain C. Ghigo J. M. Amorena B. Penades J. R.. (2003). SarA and not sigmaB is essential for biofilm development by *Staphylococcus aureus*. Mol. Microbiol. 48, 1075–1087. 10.1046/j.1365-2958.2003.03493.x, PMID: 12753197

