# Supplementary material for: Phage Lytic Protein LysRODI Prevents Staphylococcal Mastitis in Mice
Source: Front Microbiol. 2020 Jan 23;11:7. doi: 10.3389/fmicb.2020.00007 (PMC6989612; doi:10.3389/fmicb.2020.00007)
Supplement: Supplementary file 1 [file Data_Sheet_1.PDF]

Supplementary material.

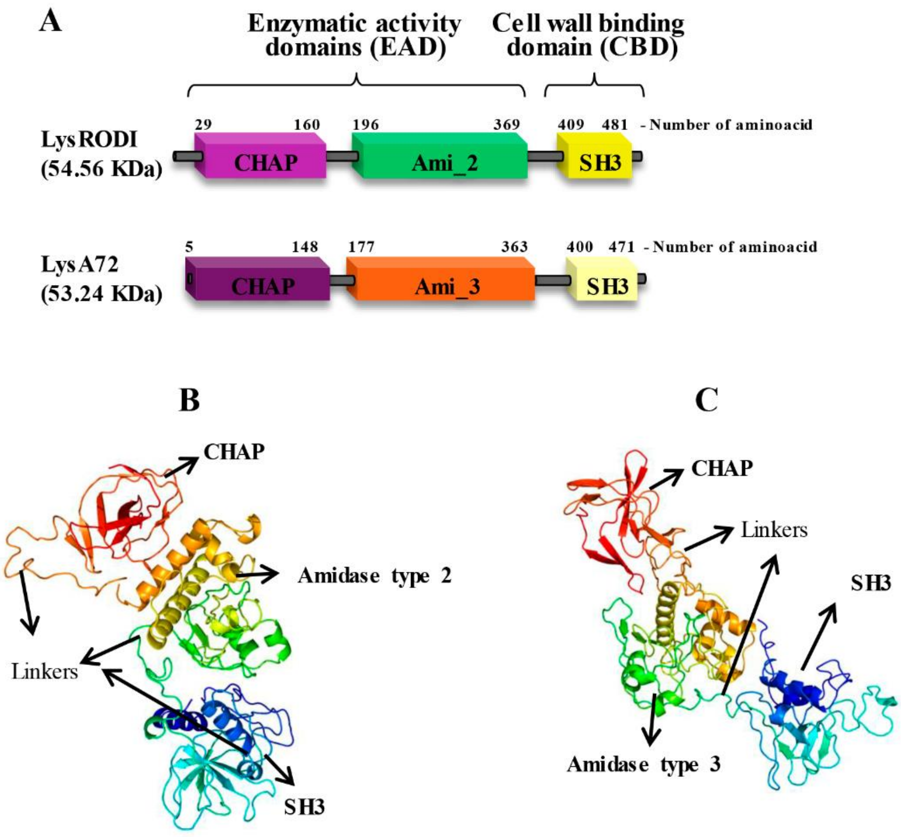

Figure S1. Bioinformatics analysis of the protein sequence of LysRODI and LysA72. A) Modular domain structure of the proteins. Both possess two catalytic domains that degrade the peptidoglycan (CHAP and Amidase) and one cell wall binding domain (SH3b) that recognizes and bind to the peptidoglycan. The amidase domain from LysRODI belongs to the type 2, while the domain from LysA72 belongs to the type3. Domains are connected by linkers of different length. Predicted models of LysRODI (B) and LysA72 (C) are presented as a rainbow colored sequence from the N to the C terminus of the protein. The high quality images were obtained using computer software Phyre 2.

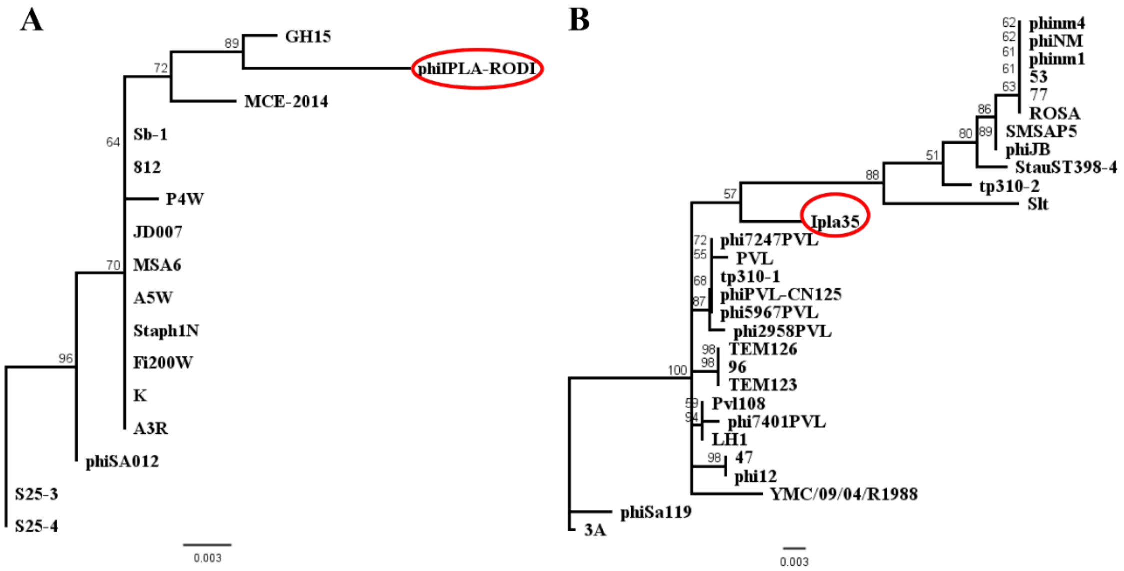

Figure S2. Clustering of endolysins based on protein similarity to A) LysRODI and B) LysA72. The dendrogram was constructed using the Jukes-Cantor model after the protein sequences have been aligned using ClustalW. The resulting similarity matrix was clustered using the Neighbor-Joining method and 1000 subsamples were generated for bootstrap analysis. The names of the branches indicate the name of the phage that encodes the endolysin. Numbers of the branch represent the percentage of consensus support. LysRODI (phage phiIPLA-RODI) and LysA72 (phage phiIPLA35) are encircled in red in figure A and B, respectively. Scale bar represents the distance between endolysins.

Table S1. Comparison of the efficacy induced by different treatments (at 0.5, 1 or 3 ×MIC) of recombinant LysRODI or CHAPSH3b proteins, in zebrafish (n=18) infected intraperitoneally with ~10^5 CFU/fish of *S. aureus*, 1 h before treatment. One group of infected zebrafish (n=18) untreated was used as control. The number of survival zebrafish were recorded at 24, 48 and 72 h post-treatment and transformed as the percentage of cumulative survival zebrafish.

**Treatment**	**Dose**	**P values by Log-Rank test [a]**		
		***vs* control**	***vs* doses**	***vs* proteins[b]**
**LysRODI**	**0.5 × MIC**	0.040	*vs* 1 × MIC: 0.29	0.956
	**1 × MIC**	0.0032	*vs* 3 × MIC: 0.96	0.964
	**3 × MIC**	0.0022	*vs* 0.5 × MIC: 0.25	0.2994
**CHAPSH3b**	**0.5 × MIC**	0.032	*vs* 1 × MIC: 0.27	---
	**1 × MIC**	0.0022	*vs* 3 × MIC: 0.30	---
	**3 × MIC**	0.0001	*vs* 0.5 × MIC: 0.041	---

[a] Log-Rank test was used to compare the % cumulative survival zebrafish in each zebrafish group (see Kaplan-Meier in Figure 3B); [b] LysRODI vs CHAPSH3b administered at equivalent dose.
